# Optimization of the Nitinol Framework of an Aortic Valve Bioprosthesis Using Numerical Simulation

**DOI:** 10.17691/stm2026.18.4.02

**Published:** 2026-08-31

**Authors:** S.V. Vladimirov, Yu.M. Prikhodko, V.V. Khakhalkin, V.P. Borodin, A.B. Mochalova, A.V. Bogachev-Prokofiev

**Affiliations:** Junior Researcher, Bioprosthetics Laboratory, Institute of Experimental Biology and Medicine; Academician E.N. Meshalkin National Medical Research Center, Ministry of Health of the Russian Federation, 15 Rechkunovskaya St., Novosibirsk, 630055, Russia; PhD, Senior Researcher, Bioprosthetics Laboratory, Institute of Experimental Biology and Medicine; Academician E.N. Meshalkin National Medical Research Center, Ministry of Health of the Russian Federation, 15 Rechkunovskaya St., Novosibirsk, 630055, Russia; PhD, Junior Researcher, Biomedical Technologies Laboratory, Institute of Experimental Biology and Medicine; Academician E.N. Meshalkin National Medical Research Center, Ministry of Health of the Russian Federation, 15 Rechkunovskaya St., Novosibirsk, 630055, Russia; Engineer, Bioprosthetics Laboratory, Institute of Experimental Biology and Medicine; Academician E.N. Meshalkin National Medical Research Center, Ministry of Health of the Russian Federation, 15 Rechkunovskaya St., Novosibirsk, 630055, Russia; Junior Researcher, Bioprosthetics Laboratory, Institute of Experimental Biology and Medicine; Academician E.N. Meshalkin National Medical Research Center, Ministry of Health of the Russian Federation, 15 Rechkunovskaya St., Novosibirsk, 630055, Russia; MD, DSc, Director of the Institute of Circulatory Pathology; Academician E.N. Meshalkin National Medical Research Center, Ministry of Health of the Russian Federation, 15 Rechkunovskaya St., Novosibirsk, 630055, Russia

**Keywords:** aortic heart valve prosthesis, numerical simulation, finite element method, hydrodynamic study

## Abstract

The application of numerical analysis methods when developing novel heart valve bioprostheses is an integral stage of their design; it enables to optimize the bioprosthetic construction and accelerate their development process.

**The aim of the study** was to conduct hydrodynamic tests of a prototype of a self-expandable transcatheter aortic valve bioprosthesis and, based on the findings, using numerical simulation, improve the geometry of the bioprosthetic nitinol frame.

**Materials and Methods:**

The study investigated an aortic valve bioprosthesis with a self-expanding nitinol frame and the leaflet apparatus of three biological leaflets fixed to the frame through the holes in the commissural posts. The bioprosthesis was tested on a test bench to evaluate the hydrodynamic characteristics of heart valve prostheses. A computer model of the valve frame was created for a finite element analysis in the COMSOL Multiphysics software environment, and validated based on the observed deformations of the bioprosthesis in a full-scale experiment. The obtained model was used for parametric optimization of the commissural post geometry to reduce their deformities.

**Results:**

Bench hydrodynamic tests revealed significant deformities of the bioprosthetic commissural posts (bending up to 5 mm) in leaflet closure under diastolic pressure. The numerical simulation using the finite element method enabled to precise the load on the posts (1.3 N instead of 1.52 N according to preliminary calculations). Based on the results obtained, an optimized frame design was developed with increased width and thickness of the beams in the area of commissural posts (from 0.3 to 0.5 mm and from 0.4 to 0.5 mm, respectively). Finite element analysis showed the suggested modification to significantly increase the structural stiffness, reducing the deformation value to 0.7 mm under the load of 1.3 N.

**Conclusion:**

The suggested approach to the application of numerical simulation demonstrated its effectiveness for optimizing the design of the nitinol frame of a transcatheter aortic valve bioprosthesis. The suggested modification of the commissural post geometry significantly reduced their deformity under the hydrodynamic load on the valve, which contributed to the preservation of the leaflet coaptation, reduced the risk of paravalvular regurgitation and prosthesis dislocation. The findings demonstrate the promise of using numerical simulation methods at a bioprosthetic design stage enabling to reduce the number of necessary physical prototypes and accelerate the development process.

## Introduction

The frameworks of self-expanding bioprosthetic heart valves are traditionally made from nitinol tubing using precision laser cutting followed by the stages of thermoforming, grinding, and electrochemical polishing. A thermoforming process is carried out in several stages, gradually expanding the cut blank of the framework to its designed shape, with each stage requiring the use of a specially designed high-precision fixing matrix. Subsequently, a valvular apparatus made from preserved biological tissue is fixed on the resulting framework using a seam material. Thus, the complete production cycle of the prosthesis — from computer model to a final prototype ready for testing — is a long-term and costly process. Additionally, when creating bioprostheses with a new design, which have no experimental evaluation, it is challenging to predict whether the developed prototypes will possess the required mechanical properties. To reduce the duration and labor intensity of development, the use of numerical simulation is a promising method. It enables to reduce design time due to the possibility to assess the functionality of new structures without the need to create numerous intermediate prototypes [1].

Currently, when developing self-expanding bioprosthetic aortic heart valves using finite element analysis, it is possible to numerically assess hemodynamics, the compliance of vessel walls, and the structure of the implant frame, as well as the functioning of the leaflet mechanism [2–4]. Although earlier studies on this topic focused only on basic modeling principles [5–6], the advancements in computational models have enabled a comprehensive evaluation of the characteristics of the designed prostheses including the strength analysis of their components, prediction of regurgitation levels, and the assessment of fatigue failure risk. It has ultimately enabled to increase the reliability and effectiveness of the designed products by optimizing the geometry and selecting materials for leaflets [7].

In the studied design of the aortic valve bioprosthesis, one of the key elements of the frame is the commissural posts, the mobility of which cannot be overlooked. In the works [8–9], the researchers modeled the stress-strain state of the leaflet mechanism during hydrodynamic impact and demonstrated the mobility of the posts to be an important factor, which has an effect on the valve performance.

Currently, despite a wide variety of works, there is no unified standard or methodology for numerical simulation of valve prostheses. This is due to the unique shapes and designs of bioprostheses, as well as the algorithms and approaches most suitable for each case. Thus, further development of new approaches to modeling bioprostheses with movable commissural posts is a relevant task, and its solution will enable to expand the field of knowledge regarding the design of such structures.

**The aim of the study** was to conduct hydrodynamic testing of a prototype transcatheter self-expanding bioprosthetic aortic heart valve and, based on the findings, improve the geometry of the nitinol framework of a bioprosthesis through numerical simulation.

## Materials and Methods

### Study subject

**Figure 1.**
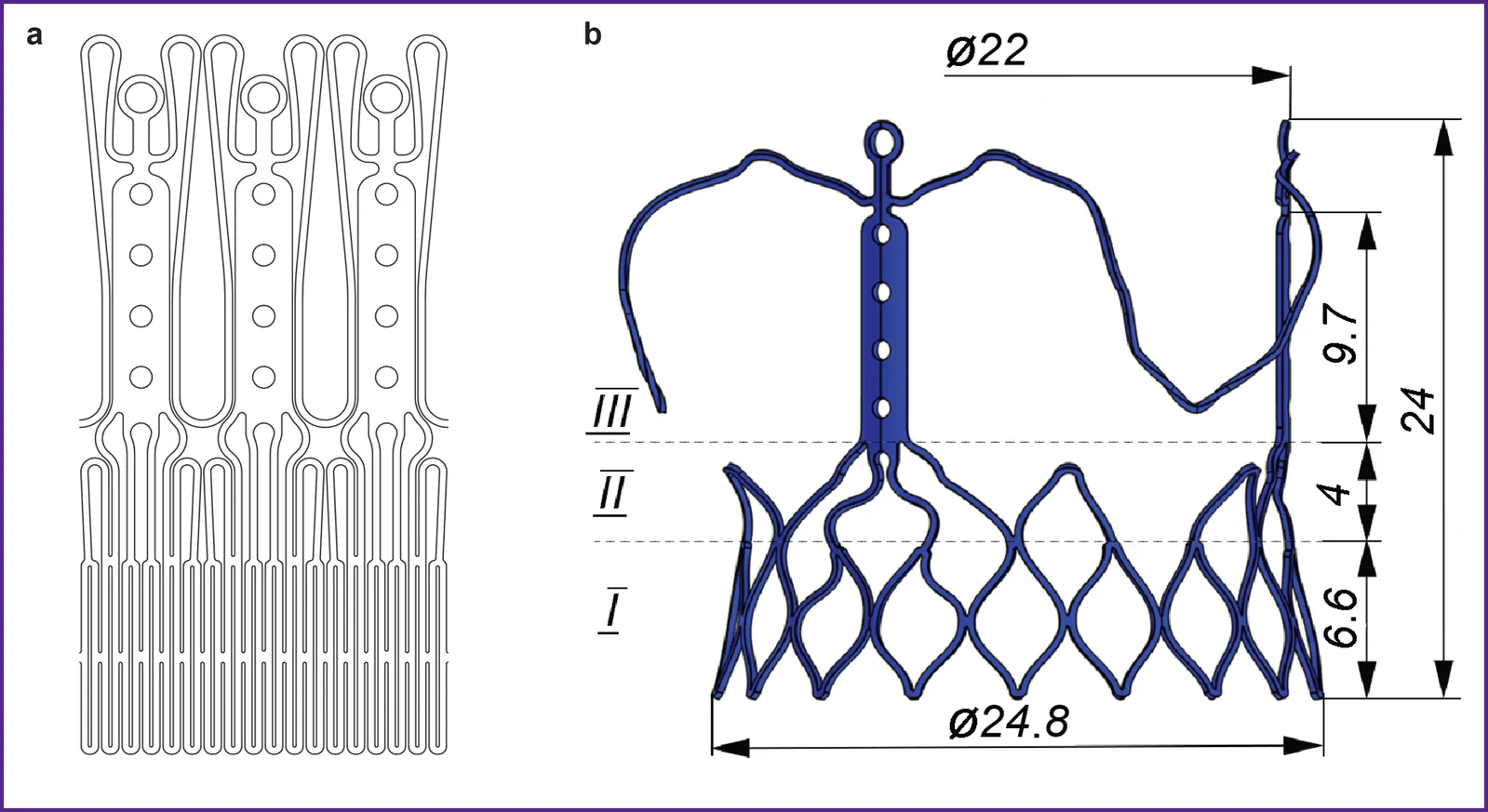
Image of the framework of the aortic valve bioprosthesis: (a) contour for laser cutting; (b) model of the valve framework in the open position

The study investigated a bioprosthetic transcatheter aortic valve, 22 mm in diameter, manufactured according to a patented technology [10]. It has a self-expanding nitinol frame (Figure 1) and a leaflet apparatus made of three biological leaflets, each 0.3 mm thick, made from pericardium.

The frame design is divided into three functional zones (Figure 1 (b)). The proximal zone (I) is shaped like a truncated cone and anchors the frame due to the radial forces generated in the left ventricular outflow tract. The middle zone (II) of the prosthesis is positioned at the level of the native aortic valve. Radial forces in this zone arise from an increase in diameter by 10–15% relative to the fibrous ring. This zone also contains outwardly directed arches preventing the valve from migrating into the left ventricular area. The distal zone (III) is formed by three commissural posts with openings for attaching the leaflet apparatus and three leaflet fasteners mimicking the anatomy of the Valsalva sinuses.

### Bench testing

The bioprosthesis was tested in a stand designed to evaluate the hydrodynamic characteristics of heart valve prostheses (MedIntell LLC, Russia) at a pressure of 90±10 mm Hg applied to the closed leaflets, in accordance with GOST 26997—2003. The working fluid used in the stand was a saline solution at 37±1°C. The support ring for the bioprosthesis was made of silicone, ensuring 10% compression of the bioprosthesis compared to its unloaded state. The functioning of the valve prosthesis was visually monitored using the camera integrated into the stand equipment (resolution 1280×720, 30 frames/s). By creating still frames from the video recording of the testing process, we selected the frames corresponding to the fully closed and fully open valve states, on which the degree of displacement of the posts was assessed.

### Numerical simulation

For the numerical analysis, we applied COMSOL Multiphysics software package (COMSOL AB, Sweden). The geometric model was constructed in SolidWorks (Dassault Systemes, France) and imported into COMSOL Multiphysics. The built-in superelastic model by Auricchio was used to describe the nitinol mechanics; the model taking into consideration the phase transformations and the nonlinear deformation behavior of the material.

The finite element mesh consisted of 38,575 second-order tetrahedral elements; the local mesh was refined in the area of the commissural posts to enhance the accuracy of geometry representation in stress concentration zones. The proximal zone I (see Figure 1 (b)) of the frame was defined as rigidly fixed around its entire circumference, simulating a tight contact with the aortic fibrous ring and preventing its displacement under load. The load was applied to the external surface of the commissural posts in the form of uniformly distributed pressure simulating the effect from the leaflets during the valve closing phase. The problem was solved within the framework of a static nonlinear analysis considering both geometric and material nonlinearities that enabled to properly reproduce the superelastic properties of nitinol.

### Load assessment

To establish the numerical simulation task, it was necessary to determine the magnitude of the force acting on the posts through the valve leaflets. For this purpose, an approximate calculation was performed based on the principles of hydrostatics and mechanics. The hydrostatic force acting on the valve leaflets during a diastolic phase was determined as the product of the pressure acting on the closed valve leaflets and the area of the projected surface of the leaflets onto a plane perpendicular to the commissural posts. Considering that the pressure differential during the bench tests in diastole is Δ*P*=90 mm Hg (11.99 kPa), and the sought area is that of a circle with a diameter equal to the valve diameter (22 mm), and amounts to *A*=3.8·10^–4^ m^2^, we can obtain the total force: *F_total_*=Δ*P*·*A*=4.56 N.

It is assumed that this force is fully transmitted to 3 commissural posts of the valve. Therefore, the load on one post is *F=F_total_*/3=1.52 N.

The obtained value is in good agreement with literature data. According to a study [11], the forces acting on the aortic valve during a diastolic phase range from 4.2 to 6.7 N depending on the aortic ring size (19–23 mm). In the work [12], the authors estimated the peak retrograde force at 6.01 N.

Considering the calculated value compliance (4.56 N) with the literature data, it was decided to use this force value as a boundary condition in the numerical simulation of the valve mechanical behavior.

The nitinol frame behavior was described using the built-in phenomenological model of superelasticity (Auricchio) in COMSOL. The material properties were defined using the following standard parameters from COMSOL database:

austenite Young’s modulus (*E_A_*) — 55 GPa;
martensite Young’s modulus (*E_M_*) — 46 GPa;
Poisson’s ratio — 0.33;
specific heat at constant pressure — 400 J/(kg·K);
density — 6500 kg/m^3^;
martensite start temperature (*M_s_*) — 245 K;
martensite finish temperature (*M_f_*) — 230 K;
austenite start temperature (*A_s_*) — 270 K;
austenite finish temperature (*A_f_*) — 280 K;
slope of the phase transition stress-temperature dependence (β) — 7.4 MPa/K;
maximum transformation strain (ε*_tr_*, _max_) — 0.056.

These values correspond to medical nitinol, which can undergo deformation of up to 8% without failure [13, 14].

The values of critical phase transition stresses at 37°C (310 K) can be calculated from the specified values based on the mathematical formulas of the Auricchio model [15–17]:

σMs=βT−Ms+Af2+σ0=458.8 MPa;

σMf=σMs+β(Ms−Mf)=569.8 MPa;

σAs=βT−Ms+Af2−σ0=199.8 MPa;

σAf=σAs+β(Ms−Mf)=88.8 MPa,

where *T* is the temperature used in tests.

## Results

### Testing on the hydrodynamic stand

**Figure 2.**
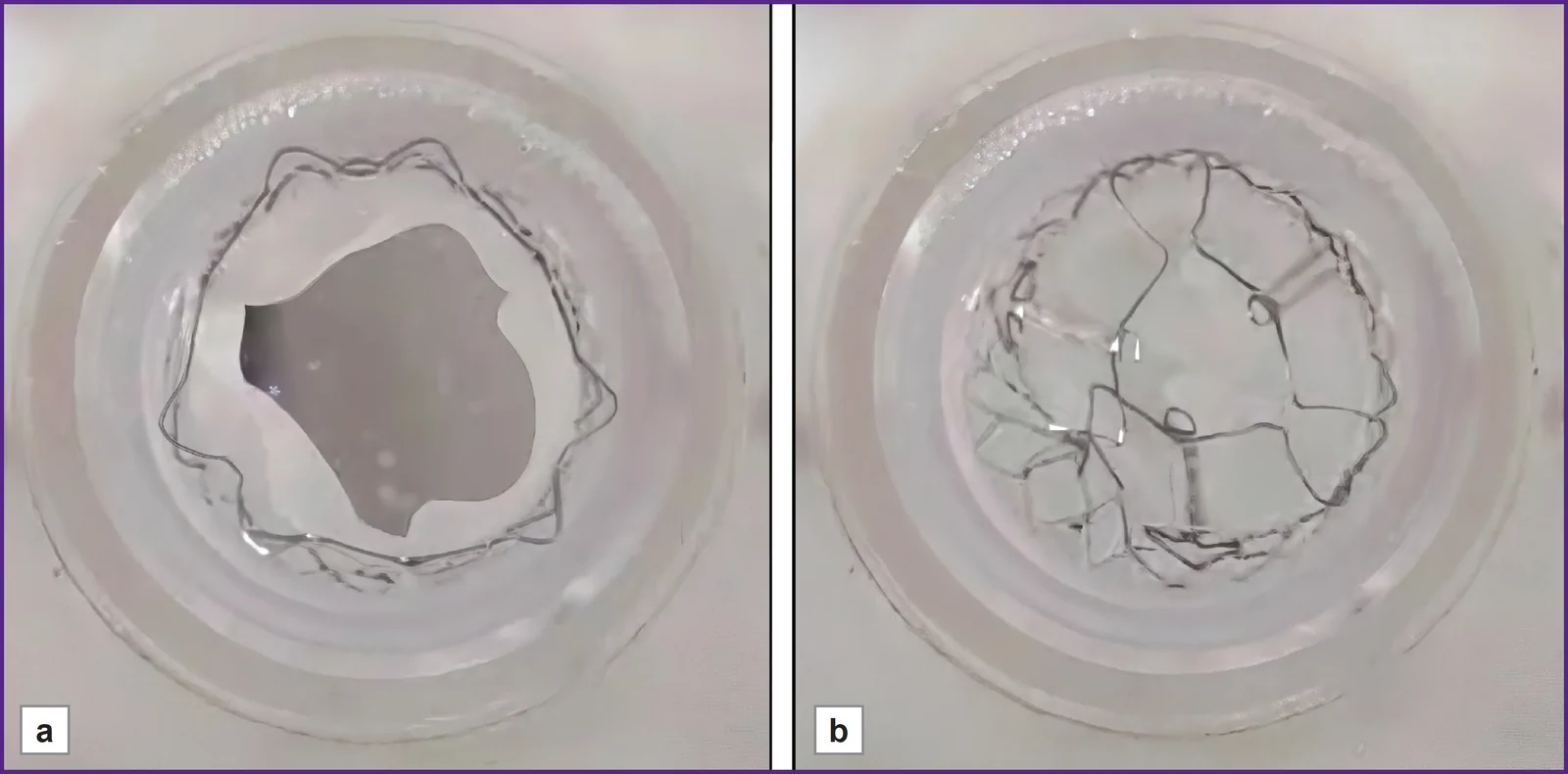
Images of the deformation of the bioprosthesis framework during testing on a hydrodynamic stand, end view: (a) open leaflets; (b) closed leaflets

The bioprosthesis testing on the hydrodynamic stand revealed the significant bending of the commissural posts under the back pressure. Video recording showed that when the leaflets were open, the commissural posts of the framework were in a normal position, parallel to the axis of symmetry of the valve, corresponding to the systolic phase with minimal load on the valve (Figure 2 (a)). However, in leaflet closed in the diastolic phase, a pronounced shift of the posts toward the center of the channel was observed, estimated at 5±0.5 mm in their distal portion (Figure 2 (b)). This shift clearly indicates the excessive compliance of the framework and its insufficient stiffness leading to the distortion of the leaflets coaptation surface and, consequently, potential regurgitation, as well as a change in the shape of the framework in its distal part (see Figure 1 (b), zone III). The identified effects necessitate a thorough analysis of the mechanical behavior of the structure. The data on the displacement served as the basis for subsequent analytical and numerical analysis.

### Numerical simulation

The observed deformities indicated that the bench tests provide only a qualitative understanding of the frame behavior, without allowing for a direct assessment of the load distribution on individual elements. To understand the bending mechanism of the posts and to verify the structural integrity, a numerical analysis considering the properties of nitinol and real loading conditions is necessary. Consequently, the COMSOL Multiphysics package was used as a tool for finite element modeling of complex nonlinear problems.

When setting the boundary conditions, the proximal part of the frame was rigidly fixed. To simulate the hydrodynamic load at the moment the valve closes, a radially directed centripetal force of F=1.52 N was applied to the top of each post.

**Figure 3.**
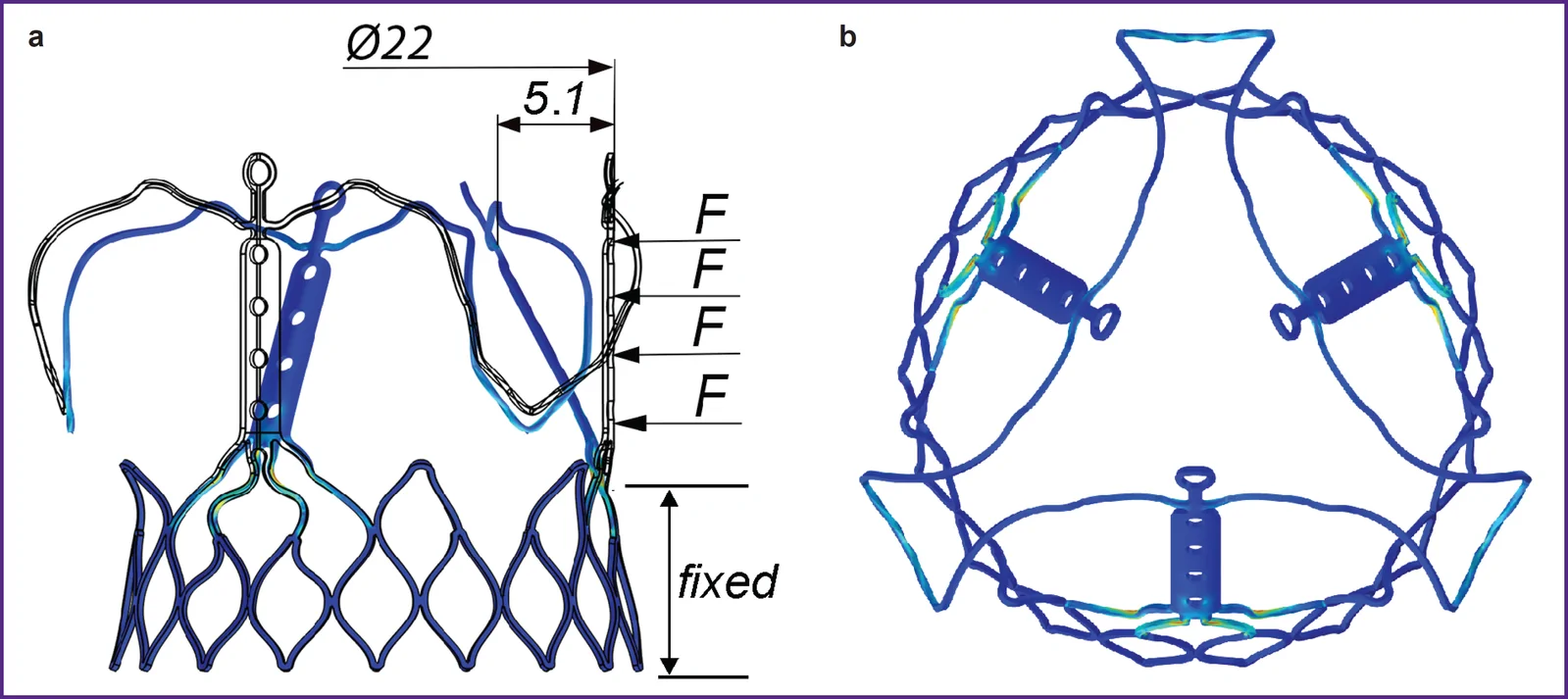
Image of the deformation of the 3D model of the bioprosthesis framework during numerical simulation before optimization Deformation of the framework under the action of force F: side view (a) and top view (b)

Upon applying the calculated force of F=1.52 N to the posts in the COMSOL model, a greater deviation towards the center was observed compared to the experiment. By adjusting the magnitude of the applied force, a value of F=1.3 N was found, at which the post deviations were approximately 5 mm (see Figure 3), being consistent with the experimental data obtained from the hydrodynamic test stand. It validates the chosen calculation methodology and, consequently, the reliability of the accepted load value.

### Frame design optimization

**Figure 4.**
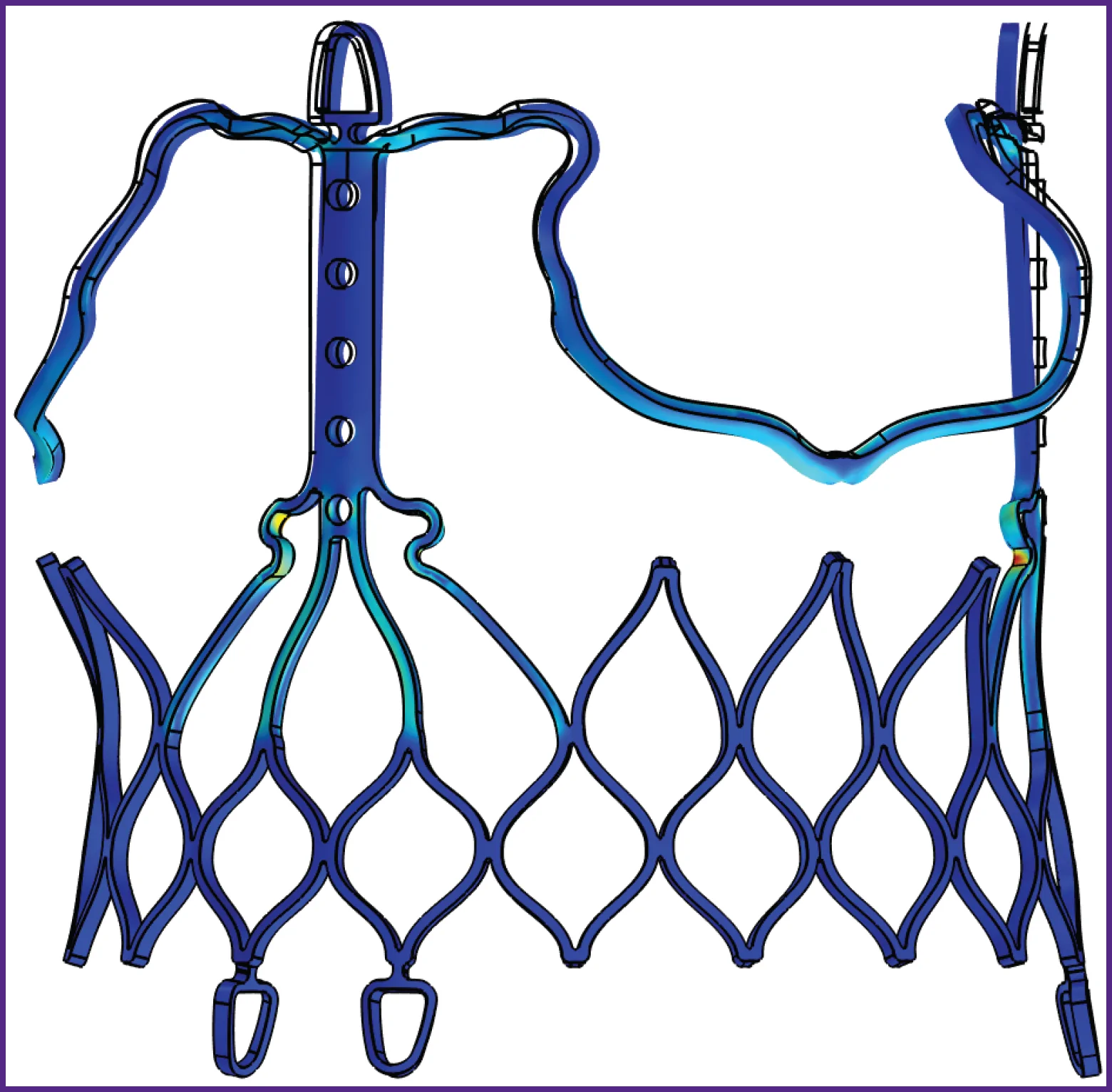
Image of the deformation of the 3D model of the bioprosthesis framework during numerical simulation after optimization

To enhance stiffness, a new design was developed with increased beam width in the post area — from 0.3 to 0.5 mm — and increased thickness of the entire frame elements — from 0.4 to 0.5 mm. The analysis using the finite element method in COMSOL showed this modification to have significantly improved the stiffness of the structure: the post deviation under the maximum load of 1.3 N was no more than 0.7 mm (Figure 4).

## Discussion

The results obtained confirm the importance of considering deformation processes in the framework when designing a transcatheter aortic valve bioprosthesis. Significant deformations of the commissural posts (up to 5 mm) observed during bench tests pose a serious issue, as they can result in compromised valve closure, the development of paravalvular regurgitation, and prosthetic dislocation — one of the most common complications following transcatheter aortic valve replacement [11, 12].

A comparison of the calculated and experimental data showed a small discrepancy in the load magnitude (1.52 vs 1.3 N), which can be attributed to simplifications in the hydrostatic model and the neglect of hydrodynamic processes during valve closure. Similar discrepancies have been noted in other studies focused on modeling the mechanics of heart valves [7, 9], highlighting the complexity of accurately reproducing prosthetic function under real conditions.

The suggested design optimization by increasing the beam width in the commissural post area and the wall thickness proved to be an effective strategy for enhancing the framework stiffness. A reduction in deformation from 5 to 0.7 mm (by 86%) indicates a significant improvement in the mechanical characteristics of the design due to the importance of commissural post stiffness to ensure the correct geometry of the valve apparatus [9].

It should be noted that increasing the wall thickness of the framework, i.e., the thickness of the original nitinol tube, may affect the framework ability to undergo radial compression to a size suitable for transcatheter delivery. However, the preliminary calculations indicate that with a wall thickness of 0.5 mm, this framework maintains sufficient elasticity enabling the use of an 18 Fr delivery system, which meets current clinical requirements [10]. Nevertheless, this aspect also requires further experimental validation.

The limitation of the present study is the simplified model of the interaction between the leaflets and the frame, since the load was applied uniformly and perpendicularly to the surface of the posts. In real conditions, the distribution of the load is more complex due to the post bending, the non-uniform deformity of the biological leaflets, and dynamic effects. Future works aim at developing a more detailed model including the interaction between the fluid and the deformable body, which will enable to more accurately reproduce the hydrodynamic processes in the valve.

In the near future we are planning to manufacture a prototype prosthesis with an optimized frame followed by testing it on a hydrodynamic stand to validate the obtained data, as well as the comparative analysis of the hemodynamic characteristics of the original and modified prostheses. Accelerated cyclic testing of the prototype with the optimized frame for fatigue strength is also on the roadmap. These studies will ultimately enable thoroughly assess the clinical potential of the suggested design and prepare it for preclinical trials.

## Conclusion

The frame of the bioprosthetic aortic valve experiences significant loads during diastole, resulting in post deviation, as confirmed by the experiments carried out on a hydrodynamic test bench. A simplified assessment of the force (1.52 N) acting on the post was found to be slightly higher than the value (1.3 N) obtained in COMSOL. Numerical simulation successfully reproduced the deformation of the posts observed experimentally. The modified frame with thicker posts demonstrated the increased stiffness and an acceptable post deviation of 0.7 mm under a similar load of 1.3 N. Further testing of the new frame on the test bench will validate the findings for actual confirmation of the design optimization.
